# Seasonality, Clinical Characteristics, and Outcomes of Respiratory Syncytial Virus Disease by Subtype Among Children Aged <5 Years: New Vaccine Surveillance Network, United States, 2016–2020

**DOI:** 10.1093/cid/ciae085

**Published:** 2024-02-15

**Authors:** Ariana P Toepfer, Justin Z Amarin, Andrew J Spieker, Laura S Stewart, Mary Allen Staat, Elizabeth P Schlaudecker, Geoffrey A Weinberg, Peter G Szilagyi, Janet A Englund, Eileen J Klein, Marian G Michaels, John V Williams, Rangaraj Selvarangan, Christopher J Harrison, Joana Y Lively, Pedro A Piedra, Vasanthi Avadhanula, Brian Rha, James Chappell, Meredith McMorrow, Heidi Moline, Natasha B Halasa

**Affiliations:** Coronavirus and Other Respiratory Viruses Division, National Center for Immunization and Respiratory Diseases, Centers for Disease Control and Prevention, Atlanta, Georgia, USA; Department of Pediatrics, Vanderbilt University Medical Center, Nashville, Tennessee, USA; Department of Pediatrics, Vanderbilt University Medical Center, Nashville, Tennessee, USA; Department of Pediatrics, Vanderbilt University Medical Center, Nashville, Tennessee, USA; Department of Pediatrics, University of Cincinnati, and Division of Infectious Diseases, Cincinnati Children's Hospital, Cincinnati, Ohio, USA; Department of Pediatrics, University of Cincinnati, and Division of Infectious Diseases, Cincinnati Children's Hospital, Cincinnati, Ohio, USA; Department of Pediatrics, University of Rochester School of Medicine & Dentistry, Rochester, New York, USA; Department of Pediatrics, University of Rochester School of Medicine & Dentistry, Rochester, New York, USA; Department of Pediatrics, Seattle Children's Hospital, Seattle, Washington, USA; Department of Pediatrics, Seattle Children's Hospital, Seattle, Washington, USA; Department of Pediatrics, University of Pittsburgh School of Medicine, and UPMC Children’s Hospital of Pittsburgh, Pittsburgh, Pennsylvania, USA; Department of Pediatrics, University of Pittsburgh School of Medicine, and UPMC Children’s Hospital of Pittsburgh, Pittsburgh, Pennsylvania, USA; Department of Pathology and Laboratory Medicine, Children's Mercy, Kansas City, Missouri, USA; Department of Pathology and Laboratory Medicine, Children's Mercy, Kansas City, Missouri, USA; Division of Viral Diseases, National Center for Immunization and Respiratory Diseases, Centers for Disease Control and Prevention, Atlanta, Georgia, USA; Department of Pediatrics, Baylor College of Medicine and Texas Children's Hospital, Houston, Texas, USA; Department of Pediatrics, Baylor College of Medicine and Texas Children's Hospital, Houston, Texas, USA; Division of Viral Diseases, National Center for Immunization and Respiratory Diseases, Centers for Disease Control and Prevention, Atlanta, Georgia, USA; Department of Pediatrics, Vanderbilt University Medical Center, Nashville, Tennessee, USA; Coronavirus and Other Respiratory Viruses Division, National Center for Immunization and Respiratory Diseases, Centers for Disease Control and Prevention, Atlanta, Georgia, USA; US Public Health Service, Rockville, Maryland, USA; Coronavirus and Other Respiratory Viruses Division, National Center for Immunization and Respiratory Diseases, Centers for Disease Control and Prevention, Atlanta, Georgia, USA; US Public Health Service, Rockville, Maryland, USA; Department of Pediatrics, Vanderbilt University Medical Center, Nashville, Tennessee, USA

**Keywords:** RSV, RSV-A, RSV-B, seasonality, hospitalization

## Abstract

**Background:**

Respiratory syncytial virus (RSV) is a leading cause of acute respiratory illnesses in children. RSV can be broadly categorized into 2 major subtypes: A and B. RSV subtypes have been known to cocirculate with variability in different regions of the world. Clinical associations with viral subtype have been studied among children with conflicting findings such that no conclusive relationships between RSV subtype and severity have been established.

**Methods:**

During 2016–2020, children aged <5 years were enrolled in prospective surveillance in the emergency department or inpatient settings at 7 US pediatric medical centers. Surveillance data collection included parent/guardian interviews, chart reviews, and collection of midturbinate nasal plus/minus throat swabs for RSV (RSV-A, RSV-B, and untyped) using reverse transcription polymerase chain reaction.

**Results:**

Among 6398 RSV-positive children aged <5 years, 3424 (54%) had subtype RSV-A infections, 2602 (41%) had subtype RSV-B infections, and 272 (5%) were not typed, inconclusive, or mixed infections. In both adjusted and unadjusted analyses, RSV-A–positive children were more likely to be hospitalized, as well as when restricted to <1 year. By season, RSV-A and RSV-B cocirculated in varying levels, with 1 subtype dominating proportionally.

**Conclusions:**

Findings indicate that RSV-A and RSV-B may only be marginally clinically distinguishable, but both subtypes are associated with medically attended illness in children aged <5 years. Furthermore, circulation of RSV subtypes varies substantially each year, seasonally and geographically. With introduction of new RSV prevention products, this highlights the importance of continued monitoring of RSV-A and RSV-B subtypes.

## Teaser

We summarize respiratory syncytial virus (RSV) surveillance during 2016–2020 at 7 pediatric medical centers and provide information on clinical severity among children with RSV-A compared with those with RSV-B and seasonal variability in dominant RSV subtype by year.

## Graphical abstract

**Graphical abstract:**
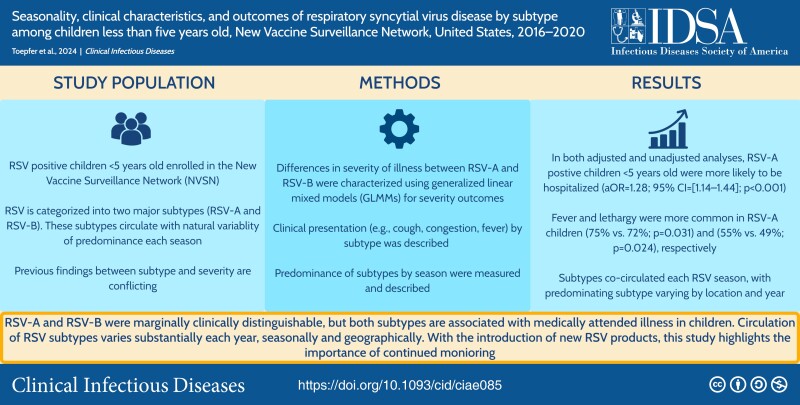
This graphical abstract is also available at Tidbit: https://tidbitapp.io/tidbits/seasonality-clinical-characteristics-and-outcomes-of-respiratory-syncytial-virus-disease-by-subtype-among-children-less-than-five-years-old-new-vaccine-surveillance-network-united-states-2016-2020-2feb8f50-35ff-4a68-ac8c-e41c08736cec?utm_campaign=tidbitlinkshare&utm_source=IO

Globally, respiratory syncytial virus (RSV) is a leading cause of acute respiratory illnesses (ARIs) in children aged <5 years, resulting in significant morbidity and mortality [1–3]. RSV-associated ARI disproportionally impacts children aged <1 year, typically accounting for over one-third of RSV hospitalizations in children aged <5 years, which has contributed to the development of monoclonal antibodies (mAbs) and maternal vaccines for children in this age group [1–3]. RSV can be broadly categorized into 2 major antigenic groups or subtypes, A and B, each comprising multiple genotypes (eg, GA1, GB1) that may vary in predominance by season and geographic location [4–6]. The glycoprotein G surface attachment protein differentiates RSV-A and RSV-B subtypes, while most neutralization activity is directed against the fusion F protein [6]. Current mAbs and vaccines in development target F proteins; however, recent studies have shown that naturally occurring binding site mutations on these proteins may differ by subtype [7]. Binding site mutations as RSV-B F protein substitutions that have the ability to reduce or increase nirsevimab susceptibility [7] may impact vaccine and prevention product rollouts.

A typical RSV season in much of the United States occurs annually from late fall through early spring, with a peak during winter [2, 8, 9]. Each season, RSV subtypes have been known to cocirculate with natural variability of subtype predominance over time in different regions of the world [10–14]. To date, clinical associations with viral subtype have been studied primarily among hospitalized children only, with conflicting findings such that no conclusive relationships between RSV subtype and severity have been established [6], although differences in clinical presentation have been reported [9]. Given our large cohort of children prospectively enrolled with RSV over multiple seasons, we are well positioned to analyze and compare the proportions of RSV-A and RSV-B subtypes, as well as their association with clinical severity. Therefore, we aim to describe seasonality, demographics (eg, age, sex, underlying conditions), palivizumab use, symptomology (eg, fever, cough), and severity (eg, hospitalization, intensive care unit admission, intubation) by RSV subtype by analyzing epidemiologic and laboratory information collected in the New Vaccine Surveillance Network (NVSN) in 2016–2020.

## METHODS

### Study Design and Population

From 1 December 2016 to 31 March 2020, children were enrolled in prospective, active surveillance in the emergency department (ED) or inpatient (IP) settings at 7 US pediatric medical centers: Cincinnati Children's Hospital Medical Center (Cincinnati, Ohio), Texas Children's Hospital (Houston, Texas), Children's Mercy Hospital (Kansas City, Missouri), Monroe Carell Jr. Children's Hospital at Vanderbilt (Nashville, Tennessee), UPMC Children's Hospital of Pittsburgh (Pittsburgh, Pennsylvania), UR-Golisano Children's Hospital (Rochester, New York), and Seattle Children's Hospital (Seattle, Washington). Children were eligible for enrollment if they had an illness duration of <14 days, were enrolled within 48 hours of admission (IP only), had at least 1 qualifying ARI sign or symptom (eg, cough, fever, nasal congestion), were wheezing, or had an apparent life-threatening event or brief resolved unexplained event. For this substudy, we limited our analyses to children aged <5 years [8]. Children were excluded if they had a known nonrespiratory cause for hospitalization, had fever and neutropenia from chemotherapy, were admitted <5 days after a previous hospitalization, were transferred from another hospital after an admission of >48 hours, were a newborn who had never been discharged home from the hospital, or had previously been enrolled in this study <14 days before their current visit or hospitalization [8]. Across all hospitals, surveillance staff enrolled children no fewer than 5 days per week in the IP setting and no fewer than 4 days per week in the ED.

### Data and Specimen Collection

Data were collected through a standardized form that included a parent or guardian interview to obtain demographic information, symptomology, and patient history, followed by a medical chart review that captured clinical markers of illness severity at the time of visit and hospitalization. During enrollment, midturbinate nasal plus/minus throat swabs were collected from children. All swabs were systematically tested for a standard set of pathogens, including RSV-A and RSV-B subtypes, using reverse transcription polymerase chain reaction [8].

### Ethics

Informed consent was obtained from a parent or legal guardian of each eligible child prior to any study procedures (eg, standardized parent or guardian interview; medical chart review; and collection, testing, and storage of respiratory specimens). Assent from eligible children was obtained at each site according to local regulations. The surveillance protocol was reviewed and approved by the institutional review boards at the Centers for Disease Control and Prevention and each of the 7 study sites (45 C.F.R. part 46; 21 C.F.R. part 56) [8].

### Statistical Analyses

We restricted analyses to children aged <5 years with a positive RSV result in surveillance testing, with molecular subtype testing for RSV-A and RSV-B from multiplex respiratory pathogen testing. Descriptive statistics were summarized as frequencies and percentages. RSV-A and RSV-B were compared using the Pearson *χ*^2^ test for categorical variables and the 2-sample *t* test with unequal variances for continuous variables. Differences in severity of illness between RSV-A and RSV-B were further characterized using generalized linear mixed models for each of 5 severity outcomes: supplemental oxygen, intensive care unit [ICU] admission, intubation, extracorporeal membrane oxygenation), and number of days in hospital, which was treated as a continuous variable, adjusting for confounders (age, underlying medical conditions, study site, and prematurity) identified a priori. For binary outcomes (hospitalization and supplemental oxygen use, ICU admission, and intubation among those hospitalized), a logistic link was used to estimate odds ratios and extract 95% confidence intervals (CIs). Among those hospitalized, length of stay was treated continuously, and the identity link was used to estimate mean differences and corresponding 95% CIs (the Kenward–Roger method was used to estimate the degrees of freedom for tests of fixed effects). Fixed effects in all models included RSV subtype (A or B); restricted cubic splines of age with 3 knots placed at the 10th, 50th, and 90th percentiles (to account for nonlinearity); and the presence of underlying medical conditions. Study site was included as a random effect in all models to account for site-related variability. Sensitivity analyses were performed for the same outcomes among children aged <1 year, with prematurity included as an additional fixed effect. All analyses were performed using SAS software (version 9.4; SAS Institute) or R (version 4.3.0; R Foundation).

## RESULTS

### Population and Clinical Characteristics

Of the 13 110 (IP) and 13 227 (ED) children aged <5 years enrolled from 1 December 2016 to 31 March 2020, 4123 (31%) and 2275 (17%) tested positive for RSV, respectively. Among those, 3424 (54%) were RSV-A and 2602 (41%) were RSV-B. A total of 372 (5%) were untyped or inconclusive or both subtypes were detected (Table 1).

**Table 1.** Clinical Characteristics of Respiratory Syncytial Virus (RSV)–Positive Children Aged <5 Years in Inpatient or Emergency Department, Stratified by RSV Subtype, New Vaccine Surveillance Network, 2016–2020

Characteristic	RSV, N = 6398^a^	RSV-A, n = 3424	RSV-B, n = 2602	*P* Value^b^
Age at screening, median (interquartile range), m	9.0 (3.0–19.0)	9.0 (3.0–20.0)	8.0 (3.0–18.0)	**.002**
Age group at screening, n (%), m				
0–5	2485 (38.8)	1318 (38.5)	1057 (40.6)	
6–11	1227 (19.2)	638 (18.6)	526 (20.2)	
12–23	1394 (21.8)	749 (21.9)	543 (20.9)	
24–59	1292 (20.2)	719 (21.0)	476 (18.3)	
Male, n (%)	3572 (55.8)	1902 (55.5)	1451 (55.8)	.87
Race and Hispanic origin, n (%)				.068
Hispanic	1516/6343 (23.9)	788/3397 (23.2)	653/2578 (25.3)	
Non-Hispanic White	2630/6343 (41.5)	1402/3397 (41.3)	1073/2578 (41.6)	
Non-Hispanic Black	1559/6343 (24.6)	897/3397 (26.4)	611/2578 (23.7)	
Non-Hispanic other	638/6343 (10.1)	310/3397 (9.1)	241/2578 (9.3)	
Risk factors, n (%)				
Breastfeeding history,^c^ n (%)	4403/5754 (76.5)	2352/3059 (76.9)	1774/2376 (74.7)	.057
Supplemental oxygen at home, n (%)	101/6395 (1.6)	45/3423 (1.3)	51/2600 (2.0)	**.047**
Ventilator support at home, n (%)	37/6393 (0.6)	10/3420 (0.3)	25/2601 (1.0)	**<.001**
Daycare, preschool, or school attendance, n (%)	2325/6388 (36.4)	1294/3418 (37.9)	876/2599 (33.7)	**<.001**
Premature birth,^d^ n (%)	970/5106 (19.0)	530/2705 (19.6)	391/2126 (18.4)	.29
Palivizumab use,^d^ n (%)	228/4533 (5.0)	112/2383 (4.7)	102/1903 (5.4)	.32
Underlying medical condition, n (%)				
≥1 underlying medical condition,^e^ n (%)	1114 (17.4)	590 (17.2)	457 (17.6)	.74
Neuromuscular condition, n (%)	187 (2.9)	87 (2.5)	87 (3.3)	.065
Immunocompromised, n (%)	61 (1.0)	38 (1.1)	21 (0.8)	.24
Respiratory condition, n (%)	573 (9.0)	307 (9.0)	227 (8.7)	.74
Hospitalized, n (%)	4123 (64.4)	2277 (66.5)	1658 (63.7)	**.025**

Bolded values signify that are a *P* value was statistically significant.

Abbreviation: RSV, respiratory syncytial virus.

^a^RSV-positive results included RSV-A, RSV-B, untyped, inconclusive, or dual detections. The 372 untyped, inconclusive, or dual detection specimens were removed from subtype-specific analysis.

^b^Comparing RSV-A and RSV-B using the Pearson *χ*^2^ test for categorical variables and the 2-sample *t* test with unequal variances for continuous variables.

^c^Denominator for breastfeeding history was restricted to children aged <3 years.

^d^Denominator for premature birth and palivizumab use was restricted to children aged <2 years.

^e^Underlying medical conditions include congenital heart malformation or other heart condition, transplant recipient, cancer, sickle cell anemia, cerebral palsy, seizure disorder or other neurologic or neuromuscular disorder, asthma, reactive airway disease, cystic fibrosis, bronchopulmonary dysplasia, chronic lung disease of prematurity or other chronic lung condition, kidney disease, Down syndrome or other genetic/metabolic disorder, blood disorders, liver disease, diabetes, chronic endocrine condition, chronic gastrointestinal disease, other developmental disabilities.

**Figure 1.**
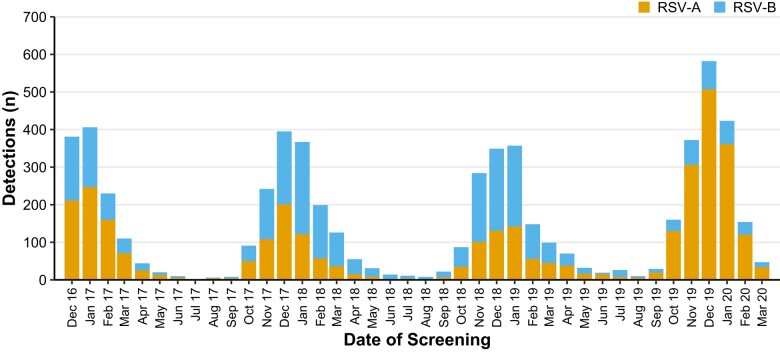
Distribution of signs and symptoms among RSV-positive children aged <5 years in inpatient or emergency department settings stratified by RSV subtype, New Vaccine Surveillance Network, 2016–2020. Fever and lethargy (but no other sign or symptom) significantly differed between groups (*P* = .031 and *P* = .024, respectively). Results presented are unadjusted for confounders. Abbreviation: RSV, respiratory syncytial virus.

Compared with children with RSV-A, children with RSV-B were younger with a median age of 8 months (interquartile range [IQR], 3.0–18.0) vs 9 months (IQR, 3.0–20.0; *P* = .023). There were no significant differences in race, ethnicity, or sex among children with RSV-A and RSV-B. Baseline home supplemental oxygen and ventilator use at home were reported more frequently among RSV-B–positive children (1% vs 2%, *P* = .047% and 0.3% vs 1%, *P* = <.001, respectively). Daycare, preschool, or school attendance was reported more frequently among RSV-A–positive children (38% vs 34%, *P* = .009). Compared with children with RSV-A, children with RSV-B had similar underlying medical conditions, breastfeeding history, and palivizumab use. Clinical presentation among RSV subtypes was similar; the most common symptoms reported during the parent or guardian interviews were cough (99%), congestion or runny nose (96%), dyspnea (85%), and irritability (85%). Both fever and lethargy were more common in RSV-A–positive children (75% vs 72%, *P* = .031) and (55% vs 49%, *P* = .024), respectively (Figure 1).

### Severity of Illness

Results from the unadjusted analysis showed that RSV-A–positive children were more likely to be hospitalized than RSV-B–positive children (67% vs 64%, *P* = .025; Table 1). The number of days of hospitalization was not significantly different between children with RSV-A and those with RSV-B (*P* = .51). Other measures of illness severity (eg, receipt of supplemental oxygen in the first 24 hours, intubation, and ICU admission) were not statistically different by RSV subtype for children aged <5 years and <1 year (Tables 2 and 3).

**Table 2.** Markers of Severity Among Hospitalized Respiratory Syncytial Virus (RSV)­–Positive Children Aged <5 Years Stratified by RSV Subtype: New Vaccine Surveillance Network, 2016–2020

Characteristic	RSV-A, n = 2277	RSV-B, n = 1658	*P* Value^a^
Days in hospital, median (IQR)	2.0 (1.0–3.0)	2.0 (1.0–4.0)	.51
Days in hospital, n (%)			
0–1	828 (36.4)	609 (36.7)	
2	556 (24.4)	418 (25.2)	
3–4	518 (22.7)	322 (19.4)	
≥5	375 (16.5)	309 (18.6)	
Clinical course, n (%)			
Intensive care unit admission, n (%)	484/2275 (21.3)	327/1657 (19.7)	.24
Supplemental oxygen use,^b^ n (%)	1449 (63.6)	1007/1657 (60.8)	.067
Intubation, n (%)	82/2275 (3.6)	69/1657 (4.2)	.37
Extracorporeal membrane oxygenation, n (%)	0/2276 (0.0)	1/1656 (0.1)	

Abbreviations: IQR, interquartile range; RSV, respiratory syncytial virus.

^a^Comparing RSV-A and RSV-B using the Pearson *χ*^2^ test for categorical variables and the 2-sample *t* test with unequal variances for continuous variables.

^b^One site reported supplemental oxygen use throughout the entire hospital stay, whereas all other sites reported supplemental oxygen use during the first 24 hours of admission only.

**Table 3.** Markers of Severity Among Hospitalized Respiratory Syncytial Virus (RSV)–Positive Children Aged <1 Year Stratified by RSV Subtype: New Vaccine Surveillance Network, 2016–2020

Characteristic	RSV-A, n = 1500	RSV-B, n = 1145	*P* Value^a^
Days in hospital, median (interquartile range)	2.0 (1.0–4.0)	2.0 (1.0–4.0)	.462
Days in hospital, n (%)			
0–1	515 (34.3)	414 (36.2)	
2	355 (23.7)	273 (23.8)	
3–4	366 (24.4)	227 (19.8)	
≥5	264 (17.6)	231 (20.2)	
Clinical course, n (%)			
Intensive care unit admission, n (%)	358/1499 (23.9)	244/1144 (21.3)	.118
Supplemental oxygen use,^b^ n (%)	944 (62.9)	701 (61.2)	.368
Intubation, n (%)	67/1499 (4.5)	62/1144 (5.4)	.263
Extracorporeal membrane oxygenation, n (%)	0/1499 (0.0)	1/1144 (0.1)	

Abbreviation: RSV, respiratory syncytial virus.

^a^Comparing RSV-A and RSV-B using the Pearson *χ*^2^ test for categorical variables and the 2-sample *t* test with unequal variances for continuous variables.

^b^One site reported supplemental oxygen use throughout the entire hospital stay, whereas all other sites reported supplemental oxygen use during the first 24 hours of admission only.

Results from multivariable analyses were largely consistent with the unadjusted results. RSV-A was associated with higher odds of hospitalization in children aged <5 years (adjusted odds ratio [aOR ] = 1.28; 95% CI = [1.14–1.44]; *P* < .001; Table 4) and those aged <1 year (aOR = 1.29; 95% CI = [1.09–1.51]; *P* = .002; Table 5). However, unlike the unadjusted analysis, RSV-A was associated with higher odds of receiving supplemental oxygen in children aged <5 years (aOR = 1.17; 95% CI = [1.02–1.34]; *P* = .023; Table 4). None of the other outcomes significantly differed between children with RSV-A and those with RSV-B.

**Table 4.** Estimates From Mixed Models for Respiratory Syncytial Virus (RSV)-A (With RSV-B as the Reference Group) Predicting Hospitalization and, Among Those Hospitalized, Supplemental Oxygen Use, Intensive Care Unit Admission, Intubation, and Length of Stay Among Children Aged <5 Years

Outcome	Odds Ratio^a^ (95% Confidence Interval)	*P* Value
Hospitalization	1.28 (1.14–1.44)	**<.001**
Supplemental oxygen use^b^	1.17 (1.02–1.34)	**.023**
Intensive care unit admission^c^	1.16 (.99–1.36)	.067
Intubation^c^	0.94 (.67–1.30)	.69
Days in hospital	−0.01 (−.28–.27)	.97

The models account for the fixed effects of age (via restricted cubic splines with 3 knots at the 10th, 50th, and 90th percentiles and the presence of underlying medical conditions. Underlying medical conditions include congenital heart malformation or other heart condition, transplant recipient, cancer, sickle cell anemia, cerebral palsy, seizure disorder or other neurologic or neuromuscular disorder, asthma, reactive airway disease, cystic fibrosis, bronchopulmonary dysplasia, chronic lung disease of prematurity or other chronic lung condition, kidney disease, Down syndrome or other genetic/metabolic disorder, blood disorders, liver disease, diabetes, chronic endocrine condition, chronic gastrointestinal disease, other developmental disabilities. Study site was included as a random effect in all models. *P* values less than a nominal value of α=0.05 are indicated in bold.

^a^Generalized linear mixed models were used to estimate odds ratios (exponentiated coefficients) for binary outcomes (ie, hospitalization, oxygen use, intensive care unit admission), and a linear mixed model was used to estimate the β coefficient for the continuous outcome (ie, length of stay).

^b^One record with a missing outcome was dropped from this complete case analysis.

^c^Three records with a missing outcome were dropped from this complete case analysis.

**Table 5.** Estimates From Mixed Models for Respiratory Syncytial Virus RSV-A (With RSV-B as the Reference Group) Predicting Hospitalization and, Among Those Hospitalized, Supplemental Oxygen Use, Intensive Care Unit Admission, Intubation, and Length of Stay Among Children Aged <1 Year

Outcome	Odds Ratio^a^ (95% Confidence Interval)	*P* Value
Hospitalization	1.29 (1.09–1.51)	**.002**
Supplemental oxygen use^b^	1.10 (.94–1.30)	.24
Intensive care unit admission^c^	1.20 (.99–1.45)	.062
Intubation^c^	0.84 (.59–1.21)	.35
Days in hospital	−0.07 (−.41–.28)	.70

The models account for the fixed effects of age (via restricted cubic splines with 3 knots at the 10th, 50th, and 90th Percentiles), presence of underlying medical conditions, and prematurity. Underlying medical conditions include congenital heart malformation or other heart condition, transplant recipient, cancer, sickle cell anemia, cerebral palsy, seizure disorder or other neurologic or neuromuscular disorder, asthma, reactive airway disease, cystic fibrosis, bronchopulmonary dysplasia, chronic lung disease of prematurity or other chronic lung condition, kidney disease, Down syndrome or other genetic/metabolic disorder, blood disorders, liver disease, diabetes, chronic endocrine condition, chronic gastrointestinal disease, other developmental disabilities. Study site was included as a random effect in all models. *P* values less than a nominal value of α = 0.05 are indicated in bold.

^a^Generalized linear mixed models were used to estimate odds ratios (exponentiated coefficients) for binary outcomes (ie, hospitalization, oxygen use, intensive care unit admission), and a linear mixed model was used to estimate the β coefficient for the continuous outcome (ie, length of stay).

^b^One record with a missing outcome was dropped from this complete case analysis.

^c^Three records with a missing outcome were dropped from this complete case analysis.

### Seasonality

**Figure 2.**
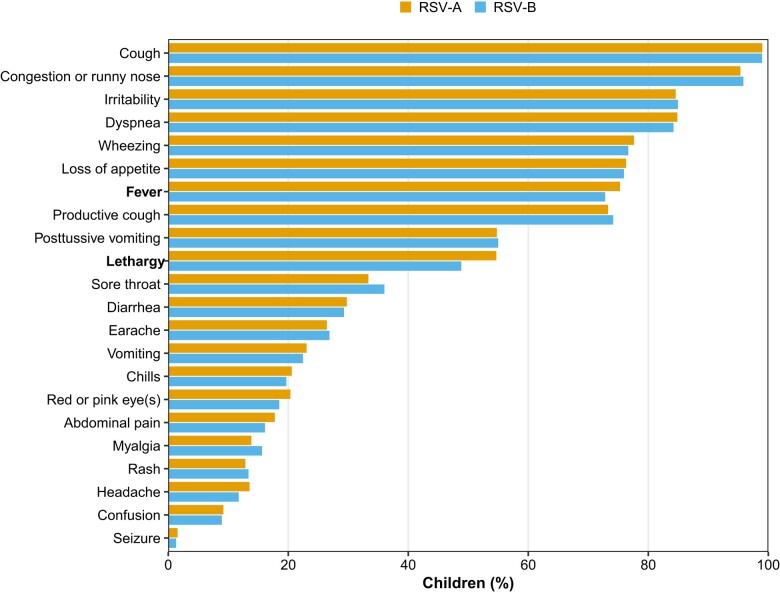
RSV detections by month and year for subtypes RSV-A and RSV-B in children aged <5 years in inpatient or emergency department settings, New Vaccine Surveillance Network, 2016–2020. Abbreviation: RSV, respiratory syncytial virus.

**Figure 3.**
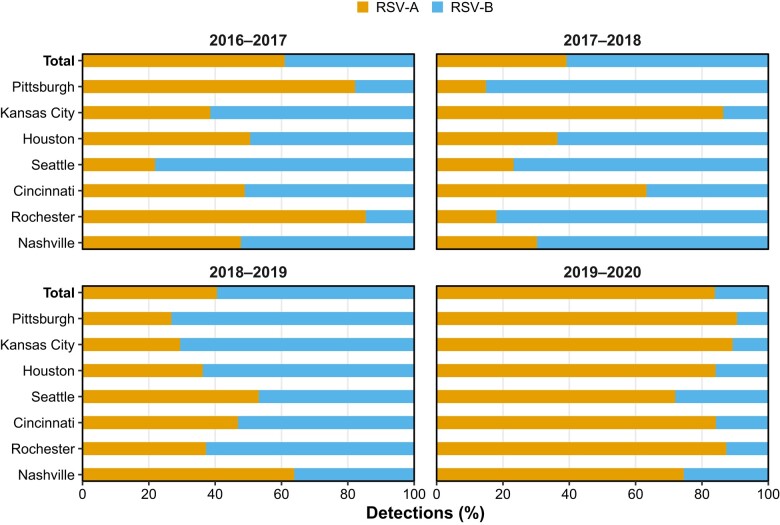
Proportions of RSV subtype detections among children aged <5 years in inpatient or emergency department settings disaggregated by surveillance season and study site, New Vaccine Surveillance Network, 2016–2020. Abbreviation: RSV, respiratory syncytial virus.

Proportions by season were as follows: December 2016–September 2017, RSV-A = 61%, RSV-B = 39%; October 2017–September 2018, RSV-A = 39%, RSV-B = 61%; and October 2018–September 2019, RSV-A = 41%, RSV-B = 59%. In contrast, 84% of RSV-positive cases aged <5 years were RSV-A during the October 2019–March 2020 season (Figures 2 and 3). The proportions among those hospitalized are shown in Supplementary Figure 1. Predominant subtypes by season occasionally differed at the local level from what was seen nationally, such as during the 2017–2018 season when RSV-B was the predominant subtype (63%), though Kansas City and Cincinnati had greater circulation of RSV-A, 86% and 63%, respectively (Figure 3). During all years analyzed, frequencies of both RSV subtypes peaked during winter. Similarly, enrollment of children with ARI in the ED and IP settings peaked during the same time of the year—late fall and early winter (Figure 2).

## DISCUSSION

In this 4-year, multicenter analysis of 6398 children aged <5 years, we observed few differences in measures of severity between children infected with RSV-A and those with RSV-B. In multivariable analyses, children with RSV-A were more likely to be hospitalized and receive supplemental oxygen. Other measures of severity did not appreciably differ by subtype. Though many studies have found that RSV-A infection may be more severe than RSV-B [15, 16], others have found evidence to the contrary or insufficient evidence of a difference [6]. Variability in findings may be due to unmeasured confounders, such as previous episodes of bronchiolitis, history of recurrent wheezing, emergence of new clades (eg, RSV-A Ontario [RSV/A/ON]), or contributions by specific genotype to disease severity [12]. Taken together, our findings indicate that RSV-A and RSV-B may only be marginally clinically distinguishable and that subtype is unlikely to impact clinical outcome. These results are in line with prior literature that reports conflicting trends of clinical impact of RSV subtype [6].

Limited differences in patient characteristics were found by subtype. Children infected with RSV-B were on average slightly younger, but the median age was only different by 1 month. Our findings are consistent with those from previous studies, suggesting that age is not a differentiating factor in terms of the need for medical attention for RSV-A or RSV-B infections in children [11, 17].

We found that RSV-A and RSV-B frequently cocirculate during the RSV season, with the predominant subtype varying by location and year. Some RSV seasons are strongly skewed toward 1 subtype, while others display greater balance. The 2016–2017 to 2018–2019 seasons were mixed, with both RSV-A and RSV-B widely circulating. However, in the 2019–2020 season, RSV-A accounted for nearly 85% of detections. Seasonality with dominance of 1 subtype, as demonstrated in this analysis of data from the NVSN, has been observed in different regions of the world [1–3, 10, 11, 13, 14]. The dominance of RSV-A during the 2019–2020 season and changes presented in the amino acid structure of RSV/A/ON recorded between 2012 and 2018 may have impacted trends in RSV circulation in subsequent years, potentially due to subtype-specific immunity [12]. Geographically diverse longitudinal studies of subtype circulation contribute to an understanding of annual and secular patterns of subtype predominance. This information might inform future development and evaluations of RSV vaccines and mAbs as circulation of subtypes with variation at critical epitopes could impact product effectiveness.

Our study has limitations. First, data were acquired through 7 academic children's health systems in the United States and may not fully represent national trends; however, the geographic breadth of study sites increases the likelihood that national patterns and regional differences were adequately captured. Second, there may be systematic differences among children enrolled and children not enrolled in the NVSN. Third, multiple years of RSV surveillance data allows for robust comparison but also resulted in large sample sizes that made even modest effect sizes statistically significant. Fourth, since most children are infected with RSV in the first 2 years of life, heterogeneous backgrounds of existing natural immunity in our study population could obscure detection of some subtype-specific clinical features of RSV infection. Last, we could not demonstrate genotype-level RSV disease associations undiscernible at the subtype level of analysis because we did not conduct viral genotyping; however, while the dominant genotypes for RSV/A/ON and RSV-B Buenos Aires continue to evolve, they historically cluster closely within these genotypes [12, 17].

This study highlights that there are subtle differences between RSV-A and RSV-B infections in young children, finding evidence of increased severity among those with RSV-A. However, both subtypes are frequently associated with medically attended illness and hospitalization in infants and young children. Furthermore, the predominance of RSV subtypes can vary substantially, both seasonally and geographically. These findings have important implications for public health policies and the rollout of RSV prevention strategies such as nirsevimab and maternal vaccination. RSV epidemiology may change post-RSV product introduction, and continued surveillance for RSV antigenic variation is warranted. The distinct patterns of RSV subtype prevalence suggest that location-specific and season-specific strategies may be necessary for effective RSV control. Further study of the clinical disease and comparative efficacy of RSV strategies against both subtypes is warranted.

## Supplementary Data

Supplementary materials are available at *Clinical Infectious Diseases* online. Consisting of data provided by the authors to benefit the reader, the posted materials are not copyedited and are the sole responsibility of the authors, so questions or comments should be addressed to the corresponding author.

## Supplementary Material

ciae085_Supplementary_Data

## Notes

***Author contributions.*** A. P. T. and J. Z. A. led the analyses and interpretation of the data, drafted the initial manuscript, and critically reviewed and revised the manuscript for important intellectual content. A. J. S. provided substantial contributions to interpretation of the data and critically reviewed and revised the manuscript for important intellectual content. L. S. S. helped supervise local data collection and critically reviewed and revised the manuscript. M. A. S., G. A. W., J. A. E., J. V. W., and R. S. provided substantial contributions to conception and design of the study and design of data collection instruments, supervised data collection locally, provided substantial contributions to interpretation of the data, and critically reviewed and revised the manuscript for important intellectual content. E. P. S., P. G. S., E. J. K., M. G. M., and C. J. H. provided substantial contributions to supervision of local data collection, interpretation of the data, and critically reviewed and revised the manuscript for important intellectual content. J. Y. L. provided substantial contributions to supervision of data collection nationally and critically reviewed and revised the manuscript for important intellectual content. V. A., P. A. P., and J. C. critically reviewed and revised the manuscript for important intellectual content. B. R. made substantial contributions to the design of the study, designed data collection instruments, supervised data collection nationally, provided substantial contributions to interpretation of the data, and critically reviewed and revised the manuscript for important intellectual content. M. M., H. M., and N. B. H. supervised data collection nationally, provided substantial contributions to interpretation of the data, and critically reviewed and revised the manuscript for important intellectual content.

***Disclaimer*.** The conclusions, findings, and opinions expressed by authors contributing to this article do not necessarily reflect the official position of the US Department of Health and Human Services, the Public Health Service, the Centers for Disease Control and Prevention (CDC), or the authors’ affiliated institutions. Use of trade names is for identification only and does not imply endorsement by the Public Health Service or the US Department of Health and Human Services.

***Financial support*.** This work was supported by the U.S. Centers for Disease Control and Prevention (CDC) (cooperative agreement RFA-IP-16-004).

## References

[1] Hall CB, Weinberg GA, Iwane MK, et al The burden of respiratory syncytial virus infection in young children. N Engl J Med 2009; 360:588–98. 19196675 10.1056/NEJMoa0804877 PMC4829966

[2] Rha B, Curns AT, Lively JY, et al Respiratory syncytial virus–associated hospitalizations among young children: 2015–2016. Pediatrics 2020; 146:e20193611. 32546583 10.1542/peds.2019-3611 PMC12874392

[3] Shi T, McAllister DA, O’Brien KL, et al Global, regional, and national disease burden estimates of acute lower respiratory infections due to respiratory syncytial virus in young children in 2015: a systematic review and modelling study. Lancet 2017; 390:946–58. 28689664 10.1016/S0140-6736(17)30938-8 PMC5592248

[4] Peret TC, Hall CB, Hammond GW, et al Circulation patterns of group A and B human respiratory syncytial virus genotypes in 5 communities in North America. J Infect Dis 2000; 181:1891–6. 10837167 10.1086/315508

[5] Pretorius MA, van Niekerk S, Tempia S, et al Replacement and positive evolution of subtype A and B respiratory syncytial virus G-protein genotypes from 1997–2012 in South Africa. J Infect Dis 2013; 208:S227–237. 24265482 10.1093/infdis/jit477

[6] Vandini S, Biagi C, Lanari M. Respiratory syncytial virus: the influence of serotype and genotype variability on clinical course of infection. Int J Mol Sci 2017; 18:1717. 28783078 10.3390/ijms18081717 PMC5578107

[7] Ahani B, Tuffy KM, Aksyuk AA, et al Molecular and phenotypic characteristics of RSV infections in infants during two nirsevimab randomized clinical trials. Nat Commun 2023; 14:4347. 37468530 10.1038/s41467-023-40057-8 PMC10356750

[8] Perez A, Lively JY, Curns A, et al Respiratory virus surveillance among children with acute respiratory illnesses—new vaccine surveillance network, United States, 2016–2021. MMWR Morb Mortal Wkly Rep 2022; 71:1253–9. 36201373 10.15585/mmwr.mm7140a1 PMC9541034

[9] Haddadin Z, Beveridge S, Fernandez K, et al Respiratory syncytial virus disease severity in young children. Clin Infect Dis 2021; 73:e4384–91. 33095882 10.1093/cid/ciaa1612 PMC8826377

[10] Yanis A, Haddadin Z, Rahman H, et al The clinical characteristics, severity, and seasonality of RSV subtypes among hospitalized children in Jordan. Pediatr Infect Dis J 2021; 40:808–13. 34260483 10.1097/INF.0000000000003193

[11] Saravanos GL, Ramos I, Britton PN, Wood NJ. Respiratory syncytial virus subtype circulation and associated disease severity at an Australian paediatric referral hospital, 2014–2018. J Paediatr Child Health 2021; 57:1190–5. 33638925 10.1111/jpc.15419

[12] Midulla F, Nenna R, Scagnolari C, et al How respiratory syncytial virus genotypes influence the clinical course in infants hospitalized for bronchiolitis. J Infect Dis 2019; 219:526–34. 30204889 10.1093/infdis/jiy496

[13] Ciarlitto C, Vittucci AC, Antilici L, et al Respiratory syncityal virus A and B: three bronchiolitis seasons in a third level hospital in Italy. Ital J Pediatr 2019; 45:115. 31462274 10.1186/s13052-019-0704-0 PMC6712785

[14] Rodriguez-Fernandez R, Tapia LI, Yang CF, et al Respiratory syncytial virus genotypes, host immune profiles, and disease severity in young children hospitalized with bronchiolitis. J Infect Dis 2017; 217:24–34. 29045741 10.1093/infdis/jix543 PMC5853407

[15] Jafri HS, Wu X, Makari D, Henrickson KJ. Distribution of respiratory syncytial virus subtypes A and B among infants presenting to the emergency department with lower respiratory tract infection or apnea. Pediatr Infect Dis J 2013; 32:335–40. 23337904 10.1097/INF.0b013e318282603a

[16] Walsh EE, McConnochie KM, Long CE, Hall CB. Severity of respiratory syncytial virus infection is related to virus strain. J Infect Dis 1997; 175:814–20. 9086135 10.1086/513976

[17] Papadopoulos NG, Gourgiotis D, Javadyan A, et al Does respiratory syncytial virus subtype influence the severity of acute bronchiolitis in hospitalized infants? Respir Med 2004; 98:879–82. 15338801 10.1016/j.rmed.2004.01.009

